# Notes on News from the Institutional World

**Published:** 1911-10-07

**Authors:** 


### NOTES ON NEWS FROM THE INSTITUTIONAL WORLD.

### THE RESULT OF THE MANCHESTER CONFERENCE

It is a fact worth noting that the British Hospitals Association has only to hold up its finger to secure an attendance of two hundred hospital representatives from England and Scotland. The special meeting on June 2, summoned as it was on such short notice, attracted to London some such number, and at Manchester last week the attendance was remarkable. In each case a definite result has been achieved. The result of the London meeting was the reception by the Chancellor of a deputation to lay before him the position of the voluntary hospitals under the proposed Insurance Bill—the first step, in other words, to secure the emendation of the offending Clause XV., which as drafted does not include hospital benefit. This was a worthy beginning. The result of the Manchester Conference has been the placing on record of the unanimous opinion of the voluntary hospitals represented that they are united in asserting that a damaging loss of income and a considerable increase in the number of in-patients will follow should the Insurance Bill pass as it stands.

### THE MANCHESTER VOTE AND THE HOUSE OF COMMONS.

Only short-sighted hospital officers will see in this an unfruitful achievement. As, however, one or two have grown with the years impatient of the passing of resolutions, let us emphasise its vital importance in this case. Sir William Cobbett, the Chairman of the Conference, no doubt voiced the sceptics when he interposed in the debate on the resolution with the remark that further discussion was unnecessary as "we are all agreed on the point." This showed that Sir William did not appreciate the urgent importance of placing on public record that agreement. It was not to agree so much as to record in public a unanimous agreement for which the Manchester Conference met. Without a resolution the decision of the Conference would have been disregarded by the public and the newspapers as the sounding of brass and the tinkling of cymbals. The real audience was not only that present in the room; it was the public outside it. To the public any repetition, argument and discussion, that led up to a resolution would have enormously increased weight. Failing the passing of a resolution, to what could the members who attended point? They are now, with the Manchester resolution behind them, in a position to urge on their respective members of Parliament a definite proposal as to how he is to vote on Clause XV. That is a force that cannot be disregarded so easily, for it can now be made known to the politicians that upwards of two hundred voluntary hospital representatives have placed on record their agreement as to the Bill. The vote of the Manchester Conference of the British Hospitals Association has given to every voluntary hospital manager throughout the constituencies a weapon that was not even forged ten days ago.

### AN ERROR CORRECTED.

In speaking at Manchester on the Hospital Saturday and Sunday Funds, Sir Henry Burdett stated that the Hospital Sunday Fund was not in a satisfactory state, and the need of the moment was to awaken the clergy and get them to understand that Hospital Sunday was the one day in the year when each clergyman and minister of religion and all the people could render a measure of personal service in the cause of the sick. In summarising the speech, several papers made these remarks refer to the Hospital Saturday Fund, an error which is serious because the statement does not apply, for this movement was never in a more vigorous or prosperous condition than it is to-day. Almost everywhere the working classes are displaying the most commendable and generous interest in the voluntary hospitals, and there are no more devoted workers than those who take an active part in promoting the cause of Hospital Saturday. The error probably crept in because, as we are advised, Hospital Sunday and Hospital Saturday in Manchester and Salford are organised by a special Hospital Saturday Fund Committee.

### THE ILL-TREATMENT OF NEWSBOYS.

Those members of the British Hospitals Association who went to the Conference at Manchester from Euston were shocked to see small boys carrying heavy trays containing books and periodicals which were supported by a piece of string slung over the shoulders, which hurt the lads, whilst the weight caused the boys to distort their bodies so that the effect must be immediate suffering and, if continued, ultimate injury to the spine, with possibly permanent deformity. If straps were supplied to the boys the evil would be lessened, but on medical, hygienic and moral grounds the use of small boys as carriers of such heavy weights is indefensible and wrong. We venture to call the attention of the directors of the London and North Western, and of any other railways where this system prevails, to the practice, with a view to its immediate discontinuance. We understand that at some stations the managers of the newspaper distributing firm who are immediately responsible for the employment of the boys recognise the evils of the system. We hope that now the attention of the public is called to the matter it will be discontinued, and never again find a place in any system for the distribution of newspapers and periodical literature.

### PERSONALITIES AT THE MANCHESTER CONFERENCE.

The type of men produced by the voluntary system was represented on many instructive sides at last week's conference of the British Hospitals Association at Manchester. If a foreigner had wanted to test the personnel of our hospitals he would have done well to attend, for all sides of English hospital life were represented. Sir William Cobbett, who took the chair, was an example of the men in public life who are not too busy to devote a large part of their time to voluntary work for the hospitals. He received his knighthood in 1909, and is equally well known as chairman of the Board of Management of the Manchester Royal Infirmary and as the head of a big firm of solicitors. Mr. Lupton was another principal speaker, whose work as treasurer for the Leeds General Infirmary is another example of that public-spirited class of rich men who give their time to hospital work. His family is a very well-known one in Leeds, possesses land in the county, and he is, we believe, also engaged in legal work.

### A THREE-SIDED HOSPITAL OFFICIAL.

Dr. Mackintosh, M.B., M.V.O., medical superintendent of the Western Infirmary, Glasgow, represents a special side of hospital life. As a qualified practitioner and institutional superintendent he has had a wide experience, and his success shows him to be a man of the rather rare type who combines a scientific training with organising power. This combination of qualities has made him traverse practically the whole field of hospital work, and some of the best-known side of that has been his advisory reports. These have been made at the request of various institutions which were beset with problems of reconstruction in an architect's and in a manager's sense. The former side of his work has gained significance from the fact that only of very recent years has hospital architecture been recognised, except by a few individuals, as a form of architecture which requires a special training. The lack of that training has produced many notorious failures in hospital building; these failures have led to certain medical superintendents giving their attention to hospital architecture. Dr. Mackintosh is the one who has done so most thoroughly, as his book on Hospital Construction proves.

### TWO INSTITUTIONAL TYPES OF WORKER.

Mr. Howard Collins, house governor of the General Hospital, Birmingham, is well known in Midland Hospital circles as an active organiser, for it was largely due to him and his committee that the recent conference of the Midland Hospitals on the Insurance Bill was held. He represents the institutional officer who realises issues which are not confined to particular or local work, and this statesmanlike view has led him to consider the political future in relation to hospitals, which will make valuable his contribution to the next Conference of the British Hospitals Association that is to be held at Birmingham next year. Mr. J. Courtney Buchanan, who read the last paper, is a younger man, though he has been engaged in hospital work between ten and twenty years. He is a hospital secretary whose keen interest has taken advantage of such new legislative proposals as the Poor Law Commission's Reports and the Insurance Bill to examine theoretically how the new tendencies will affect the hospitals. It is sufficient to ask, Can we imagine any of the younger secretaries doing this twenty-five years ago? to show the new type of men who have been attracted to this profession.

### A NOTE ON SOME MANCHESTER SPEAKERS.

Hospital officials at some time or another have to address an audience. To some it is a painful ordeal—painful alike to speakers and to listeners. These reflections are excited by a careful attention to the speakers at the Manchester meetings last week. More than one gentleman there would have commanded attention for his remarks had he paid the slightest attention to delivery. To speak slowly and to face the audience rather than the floor are two very simple rules that go a long way towards success. For those who would like to speak effectively there is, of course, a Public Speaking Club of Great Britain now existing in London. Indeed, the opening meeting of the second session took place on Friday last. Hospital secretaries, however, generally have more club meetings to atttend already than they want, and so a simpler procedure can be recommended at least to the energetic younger men. Learn to speak as the facile authors of Fabian essays learned a quarter of a century ago—and few men have gained better results from practice—by attending and speaking at every meeting in your neighbourhood. In six months your fluency will have increased enormously, and you will be less driven to avoid meetings when the subject is always different, and you have such a definite aim as facility in speaking to take you to each one.

### HOUSE COMMITTEE AND MEDICAL STAFF AT YORK COUNTY HOSPITAL.

In view of the interest excited in Dr. E. S. Reynolds' paper on the "Representation of Hon. Medical Staffs upon Boards of Management of Voluntary Hospitals," which he read at the Conference of the British Hospitals Association at Manchester, and which we hope to publish next week, it is timeous to recall that only last month four members of the honorary consulting staff were admitted ex-officio members of the House Committee of the York County Hospital. From the speech of Sir Joseph Sykes Rymer, who presided, it appears that the aim of the new rule was to pay a compliment to retired members of the staff. That being so, his tilt at the Court of Trustees for limiting the number may be discounted, since a limit alone makes the compliment of special value. He will, perhaps, find more supporters in his contention that the final control of the Court of Trustees is an antiquated and not very satisfactory one. Dr. Reynolds, of course, was at pains to show the necessity, from an administrative point of view, of having active members of the medical staff represented on the board of management.

### ONE CONSEQUENCE OF STATE AID.

We have always insisted that the only form in which the voluntary hospitals can maintain their present independent position, whilst undertaking work for the State, is to treat the matter as one of business and to undertake such duties only when the State agrees to pay pro rata for the actual work it requires the voluntary hospital to do on its behalf. To such payments must be added some 10 per cent. for the remuneration of the medical staff, and therecan be no reasonable objection to the State sending its own inspectors to report on the work thus undertaken. State aid given in the form of a dole or contribution, instead of as a payment for actual work done, would soon destroy the voluntary hospital system. The kind of thing which would then probably happen may be illustrated by the action of the member of the New Zealand Government who is the Minister for Hospitals. The Hospital Board of the Auckland Hospital appointed an English nurse trained at the Wigan Infirmary to the office of lady superintendent. The Minister has refused to approve this appointment, and much trouble has arisen in consequence from the fact that the appointment was circulated in the press before it was submitted to the approval of the Minister, who has refused his approval on the advice of the Inspector-General of Hospitals and his assistant. The New Zealand Act gives the Minister for Hospitals power of veto for twenty-one days, and the Minister contends that the officers of the Department should be in a better position to know the qualifications of the applicants for the position of matron than the local Board can possibly be. The deliberate intention of Parliament, the Minister explains, was that during these twenty-one days the officers of the Department should make representations to the Board regarding the suitability of candidates for proposed appointments. The wording of the present clause is less stringent than a former one which gave the Minister an absolute veto, but it amounts to a power vested in the Minister to advise Boards, who, it was thought, would be guided by the opinion of the departmental officers. In practice, as this case shows, the Minister does still veto these appointments, because he properly maintains that if consulted he cannot give his consent unless he is first satisfied that the Board has made the best choice that it was possible for it to make in the circumstances.

### THE HOSPITAL OFFICERS' ASSOCIATION.

The programme of the Hospital Officers' Association for the forthcoming autumn and winter session, which has just been issued, promises a series of interesting and instructive gatherings. Founded some ten years ago, the Association has from the outset proved successful, and numbers amongst its members lay officials of all grades from all the most important hospitals of London. There is also a branch in Manchester. The social events include an annual dinner, which takes place in November. The opening meeting is on October 20, when Mr. Richard Kershaw (Secretary of the Central London Throat and Ear Hospital) will deliver his presidential address. The steward's department and the duties of a secretary's clerk will be discussed later in the session, when we shall note the chief points of interest.

### STAFF CHANGES AT A LONDON EYE HOSPITAL.

By the resignation recently of Mr. T. Brittin Archer, M.R.C.S., from the position of senior surgeon to the Central London Ophthalmic Hospital, Mr. Ernest Clarke, M.D., B.S., F.R.C.S., succeeds to the appointment. Mr. Clarke is also consulting ophthalmic surgeon to the Miller Hospital. Dr. Campbell has completed his engagement as house surgeon and has returned to Canada. By the way, we are glad to learn that rapid progress is being made with the new hospital building in Judd Street, St. Pancras, the opening of which was described fully in a previous number.

### THE NEW BOARD AT WEST BROMWICH HOSPITAL.

The King Edward VII. Memorial Fund, which has been raised to free the hospital from debt and to endow a bed, amounts (to date) to £3,600. Mr. J. Norton Griffiths, M.P. for Wednesbury, has been elected president, Mr. F. T. Jefferson, J.P., treasurer, Mr. T. Foley Bache honorary secretary, and Col. G. Walton Walker, Mr. F. B. Barclay, Mr. C. Beech, J.P., Mr. J. Brockhouse, J.P. (Mayor of West Bromwich), Mr. W. T. Davies, Mr. Thos. Jones, Mr. J. Moore, Mr. T. H. Spencer, Mr. C. Thomlinson, J.P., Mr. H. Tomblin, Mr. S. N. Williams, and Mr. S. Woodhall were appointed to serve on the Board of Management for the ensuing year.

### THE OPENING OF THE SESSION AT CHARING CROSS HOSPITAL.

A spirit of hopeful enthusiasm was distinctly in evidence at the opening of the Winter Session at Charing Cross Hospital on Monday afternoon, when the annual distribution of prizes took place in the out-patients' hall. For the first time in the history of the school the President of the hospital, Princess Louise, Duchess of Argyll, gave the prizes away. Her Royal Highness was accompanied by the Duke of Argyll, and a very large audience was in attendance. An additional touch of colour was afforded this year by the wearing of academical gowns by the members of the staff and the resident officers. The chief interest in the proceedings was centred in the report of the Dean, Dr. William Hunter, who has recently devoted his time and money unsparingly in furthering the interests of the school and conducting to a successful issue the important change of policy referred to elsewhere. Extensive alterations and reorganisations of all the departments of the school were necessary as a sequel to this decision, and an opportunity was afforded to visitors to inspect the school after the meeting, when it was seen that many beautiful as well as useful improvements had been carried out by the Dean and the school council. Dr. Hunter has certainly done much to encourage everyone connected with the school to enter upon an important period of their history with fresh zeal and determination to keep in view the best interests and the full requirements of University medical education. The Duke of Argyll made an appeal for a sum of £20,000 for a fund to be used for the equipment of the school, and which would directly benefit the hospital as well. Mr. G. Verity, chairman of the Finance Committee, proposed a vote of thanks, and referred to the great difficulties they had had to contend with at the hospital; but he was convinced that the hospital was more than ever needed where it is and that its future prospects were encouraging to the devoted and enthusiastic workers with whom he was associated. Princess Louise acknowledged the vote of thanks in a short speech full of sympathy and congratulation, and thanked the Dean and his colleagues for their hard work for the school at this important juncture. A most interesting account of the foundation and progress of Charing Cross Hospital was handed to the visitors. In it is traced the history of the institution from its inception by Dr. Benjamin Golding, whose bust was given a place of honour on the platform.

### THE NEW WARD AT SOUTHPORT INFIRMARY.

Special pains were taken the other Sunday to make the opening of the new Women's Medical Ward at Southport Infirmary an impressive occasion. Being held on a Sunday a special service was arranged, the parts of which had been very carefully thought out. The fact that the giver of the ward would not allow his, or her, name to be made known gave to many of the public a curiosity to see it that they might not otherwise have felt. The new ward has been designed so as to form eventually part of a new block. Care has been taken that the children's ward should not be deprived of its proper amount of light. The building contains a total of ten beds, besides a ward-kitchen, sisters' sitting-room and store-rooms. The floors are of pitch-pine, in the annexes of terrazzo, the wards being heated by radiators, though fireplaces have not been forgotten. A steam pump secures an accelerated circulation of water. The architect is Mr. Francis P. Halsall, A.R.I.B.A., and the supervisor appointed by the board was Mr. Franklin Hilton. The total cost is expected to be about £2,500.

### CHANGES AT CHARING CROSS MEDICAL SCHOOL.

From the Report of the Dean, Dr. William Hunter, it appears that the year now ended has been an eventful one in the history of this school. The future policy of the school, with a view to increased success and additional efficiency as a constituent medical college of the University of London, has been under the careful consideration of, and the subject of detailed reports to, the council of the hospital and committee of the school during the past year. After the fullest consideration, extending to July last, the council and school decided to make an important change regarding the teaching of the primary and intermediate studies of the curriculum, and, taking advantage of the unique position of the hospital and school (within 5 minutes' walk of the Scientific Laboratories of the University of London in King's College) have made arrangements with the University of London for the teaching of these earlier studies in its laboratories. This change of policy has placed at the disposal of the school more accommodation and excellent laboratories for the final studies in pathology, bacteriology, public health, pharmacology, etc. The school has for many years been in possession of an exceptionally good and well-arranged pathological museum containing from 3,000 to 4,000 prepared specimens, and the additional laboratory provision now made will give the hospital one of the most commodious and adaptable pathological institutes attached to any school of medicine. All departments of the students' club have been renovated and greatly improved, especially the library, reading-rooms, and dining-room. In addition, a commodious department of the college, the Anatomical Museum, has been set aside for purposes of reading and study for the students while they are carrying out their earlier scientific work in the adjacent university laboratories. The arrangements made provide that the students will now pursue their earlier scientific studies at the university laboratories, gaining thereby the best scientific education which the University of London can provide, while they continue as heretofore to make use of their own Charing Cross medical college, of its library, of its reading rooms, of its club rooms, dining room, societies, etc., thus entering at once into the collegiate social life of the school. The changes inaugurated by H.R.H. Princess Louise open up a new prospect of increased usefulness and efficiency for the hospital and school as the most central teaching college of the University of London.

### A DUBLIN HOSPITAL'S JUBILEE.

A few days ago the Mater Misericordiæ Hospital, Dublin, celebrated its golden jubilee. Arising out of the good work of the pious foundress of the Order of Mercy, Catharine McAuley, the foundation stone was laid by Archbishop Cullen in September 1852. It was opened on the same day of the month and by the same Archbishop in 1861. In 1886 the west wing was inaugurated, and in 1891 its training school for the nursing staff was opened. To-day the hospital provides for 340 patients, and is happy to possess a member of its original medical staff in the person of its consulting physician, Sir Francis Cruise. The well-wishers of this institution are eagerly wondering what share it will receive of the 50,000*l.* which Lord Iveagh gave the other day to the Dublin hospitals, the distribution of which has been handed over to a committee, that includes Lord O'Brien, the Irish Lord Chief Justice. It is not yet known when the Report of this committee will be ready.

### A NEW HOSPITAL FOR COUNTY DURHAM.

Harrogate and district has just received deeds in respect of 150 acres of land, a cheque for £10,000, and scrip value £20,000 as an endowment, which will produce a total revenue of about £1,400 a year, from Mr. Richard Murray, of Elm Park, Harrogate, with which to build and found a hospital. In acknowledging the thanks of the local authorities the giver expressed the hope that once the site had been chosen local architects and contractors should be appointed to carry out the work. The hospital will probably be built at Blackpyne, and is intended to serve the districts of Blackhill, Consett, Leadgate, and Shotley Bridge. Details concerning the construction and the proposed number of beds we hope to publish later.

### MR. H. O. WILLS' HOSPITAL BEQUESTS.

It was only to be expected that in his will the late Mr. H. O. Wills would remember the Bristol hospitals in a marked manner, such enthusiastic hospital workers has his family included, from Lord Winterstoke downwards. Mr. H. O. Wills, as our readers were informed in the early part of September, died a few weeks ago, and his estate has been sworn provisionally at £2,000,000. The Bristol General Hospital receives £3,000, a similar sum being left to the Royal Hospital for Incurables, Putney. The Bristol Royal Hospital for Sick Children is left £2,000, and the British Home for Incurables at Streatham a similar amount. Mr. Godfrey Hamilton, the secretary of the National Hospital for the Paralysed, is to be congratulated on a legacy of £500, which sum is left also to the Royal United Hospital, Bath. The hospital work of the Wills family in Bristol has been alluded to often in our columns, and should set an example to every smoker of the Imperial Tobacco Company's products, from which their great wealth was derived.

### THE SIR CHARLES DILKE MEMORIAL.

The provisional committee which is supporting the scheme for providing a memorial to Sir Charles Dilke in the Forest of Dean, which he had represented in Parliament since 1892, has secured Lord Beauchamp, Lord Lieutenant of Gloucestershire, as its chairman. The committee, of which Sir William Wedderburn is deputy chairman and Mr. Harry Webb treasurer, hopes that enough support will be forthcoming for the building of a hospital. A good many people will feel that the striking personality and stormy career of the late baronet will find few less controversial and more fitting forms than a hospital for the tangible preservation of his memory.

### MEDICAL EVIDENCE IN CORONERS' COURTS.

A recent case has brought forward once again the anomaly that medical evidence is paid for in the county courts, and apparently in all other courts save the coroner's. In the first Report of the Committee of Inquiry into the Coroner's Office, which was published in 1909, Dr. F. J. Waldo, M.D., his Majesty's coroner for the City, gave evidence on this point, inclining, if we remember, to the payment of all doctors, whether in hospital cases or not. His arguments have survived the public forgetfulness of the Commission, and he would be doing good service by taking up the question again and by re-stating them in the light of recent instances. Unpaid medical evidence is becoming an increasingly sore question, and we know no one who is better entitled to write on the subject than Dr. Waldo.

### THE FLOATING HOSPITALS OF NEW YORK.

Our own exceptionally hot weather, and this year's excessive infantile mortality from diarrhœa have attracted public attention to the hospital barges with which visitors to America have become acquainted on their entry into New York harbour. These floating hospitals, which are under the charge of a certain St. John's Guild, the members of which are rich city merchants and fashionable doctors, were designed to provide a cooler environment for the New York slum children, who, in greater numbers than our own, die from the effects of heat in the capital. A typical ship has some 200 cots on the lower deck, a deck playground covered with wire netting, a room fitted up for salt-water baths, and the inevitable ice-plant which, of course, is used principally for preserving the large quantities of milk required on board. The ships are run partly in connection with the hospitals and partly in connection with the civic authorities. No infectious cases are admitted, and there are special wards for the treatment of any infectious disease that may develop. A week is, as a rule, the longest period for which a child is allowed to stay on board, and the hospital barges, for they rely on tugs for movement, are alternately going and returning. The change to a cooler atmosphere generally effects a quick cure—but how soon a return to the slums undermines the good work does not very publicly transpire.

### A SUCCESSFUL EXPERIMENT AT CAMBRIDGE.

A year ago we noted the enterprising foundation at Cambridge of a Workers' Hospital Fund, and commended the suggestiveness of the attempt to organise in the county towns a fund analogous in principle to those already prosperous in the factory and business centres of the north. During the past twelve months we have kept an interested eye on its progress and now gladly receive its first annual report. It should be remembered that the raising of £1,000 a year chiefly for the benefit of Addenbrooke's Hospital was the aim of the fund, which has been ably worked for. The net sum of £600 has been subscribed, and 106 firms have become contributors. That is a good beginning, and, according to Rule 4, which we quoted at the outset, seven tenths of this amount—that is to say, £421—are allocated to Addenbrooke's Hospital. One tenth goes to the Cambridge and District Nursing Home and the remainder is to be banked, so that it may be used at the committee's discretion for the use of members in need of sanatoria or of convalescent home treatment. It is hoped in the near future that the Fund may obtain representation on the committee of management of the hospital, and the chairman of its General Committee, Major Albert Pell, has just sent to the Fund a letter, from which we take the following: "My Committee welcome your gift not only as a most valuable increase to our finances, but also as a free and spontaneous gift from hundreds of working men who know well what Addenbrooke's means to the poorer part of the community. The response to the recent appeal for funds to enable us to build a new children's ward and out-patient department has been most gratifying; the question of increased upkeep and maintenance has, however, also to be considered, but the Committee will be largely relieved from anxiety on this score if they can rely annually on such generous help from the Workers' Hospital Fund." It is proved, in short, that workers' funds can be started successfully in the retail businesses of county towns.

### NEW PROFESSORIAL APPOINTMENTS AT GLASGOW.

ONE fact about the first holders of the four medical chairs just established and filled under the new ordinance of the University of Glasgow is that the Western Infirmary is well represented among them. Dr. Munro Kerr has been appointed to the Muirhead Chair of Obstetrics and Gynæcology, and Dr. Robert Kennedy to the St. Mungo Chair of Surgery; Dr. Walter K. Hunter to the Muirhead Chair of Medicine, and Dr. John H. Teacher to the St. Mungo (Notman) Chair of Pathology.

### PROFESSOR HUNTER.

PROFESSOR WALTER K. HUNTER, who is forty-four years of age, received his medical education at Glasgow University, where he graduated M.D. in 1897 and D.Sc. in 1901. He has taught clinical medicine for the past fifteen years—first as tutor and later as physician and lecturer in the Royal Infirmary.

### PROFESSOR TEACHER.

PROFESSOR JOHN H. TEACHER is forty-one years of age. He graduated at the University of Glasgow in Medicine in 1893, with high commendation, being placed second graduate of the year. After a year in the Western Infirmary as resident physician under Dr. Tennent, he was appointed to the Pathological Department of the Hunterian Museum, and in 1896, at the request of the late Sir William Gairdner, he went to Rio Tinto, Southern Spain, to assist Dr. R. J. Marshall in certain investigations on malaria. In May 1905 he was appointed senior assistant in pathology in the University, and at the same time assistant pathologist to the Western Infirmary and assistant pathologist to the Royal Hospital for Sick Children. It fell to him to reorganise the Pathology Department of the Royal Infirmary in temporary premises pending the erection of a new Pathological Institute.

### MASTER OF FOXHOUNDS SUCCUMBS TO HYDROPHOBIA.

CONSIDERABLE prominence has been given to the medical opinion expressed at an inquest upon the death of a Master of Foxhounds. Apparently the gentleman during the last few days of life presented symptoms which were considered to be diagnostic of hydrophobia. This opinion was strengthened by a history of the receipt of a bite from a fox, through a thick woollen glove, at the end of a run near the close of the last hunting season. It is possible that the medical opinion may be correct; yet it is perhaps to be regretted that medical opinions, which may be open to criticism, should be widely circulated in lay papers. It had been hoped that hydrophobia no longer existed in the country, and, so far as can be judged from accounts of the inquest upon the Master of the South and West Wilts Foxhounds, it it not clear that this dreadful disease is to be considered to be still with us. The disease may possibly have smouldered on amongst foxes, although dogs have for some years been free. We believe also we are right in saying that foxes are sometimes imported, but the conditions under which foxes are imported may render this source of the disease improbable. However this may be, the symptoms from which the unfortunate gentleman suffered may have had another origin. The symptoms of hydrophobia, like the early symptoms of tetanus, indicate the action of certain toxins upon some of the nuclei at the base of the brain. But similar symptoms may occur in the absence of these particular sources of irritation. The characteristic feature of tetanus is not often simulated, and acute affections likely to be mistaken for hydrophobia are also rare. But they exist, and there is nothing in the symptoms as described in the lay papers to put the view out of consideration that the case may be an example of the group of affections described under the heading "Acute glosso-labio-laryngeal paralysis."

### MORE REGULATIONS FOR DISPENSERS.

THE legislature of recent times shows a distinct tendency to intervene in the work of hospital dispensers by defining in various ways the handling of poisonous drugs. In February a new bottle indicative of ammonia is to come into force. The autumn quarter also sees the enforcement of the new regulations for the sale of mineral acids. Accordingly vitriol, or sulphuric acid, spirit of salt, or hydrochloric acid, nitric acid, and salt of lemon must only be sold by retail in bottles which are distinguishable by touch from ordinary bottles and bearing the usual warning label, to which the name and address of the seller are to be attached. According to the latest returns of the Registrar-General—those, that is, for 1909—hydrochloric acid caused 99 deaths, nitric and sulphuric acids 7 each, besides which there were many serious accidents. The multiplication of shapes may prove rather a strain on the memory of poor people, for whom, as hospital dispensers know, this paternal legislation is intended. What is the objection to a standardised bottle for all dangerous substances? It would be interesting to learn how many dispensers believe the new regulations will assist them in their work.

### THIS WEEK'S DRUG MARKET.

BUSINESS in the drug market continues fair, but there is still room for improvement. The advance in the price of sugar affects the value of a number of galenical preparations which are now listed at higher prices. An advance in the price of cocaine is expected to take place shortly. Some brands of eucalyptus oil are slightly dearer, and the winter demand for the oil has set in. Balsam of tolu is dearer. Cod-liver oil is, if anything, tending lower in value. Sugar of milk has again been advanced in price. Senega is dearer and, contrary to expectation, cream of tartar is slightly dearer. Opium, morphine and codeine are unchanged in value, but in view of the Turko-Italian war there is no likelihood that the prices of these drugs will recede in the near future.

